# Supplementary material for: Improving the pragmatic usefulness of the scoring matrix for the Consolidated Framework for Implementation Research (CFIR). A proposal for a more frequency-based approach: The CFIR-f
Source: PLoS One. 2023 Nov 30;18(11):e0295204. doi: 10.1371/journal.pone.0295204 (PMC10688659; doi:10.1371/journal.pone.0295204)
Supplement: S1 Table — (PDF) [file pone.0295204.s001.pdf]

**S1 Table. Key ideas of themes identified as barriers, enablers or neither from semi-structured interviews**

| Themes | Key ideas |
|---|---|
| **Technical, logistical and referral challenges** | *Overall complexity of the referral system*<br>The two systems principally responsible for the monitoring and filling of vacancies in FFT-CW® were noted by several policy and program experts as unwieldy in places, and complex overall. Setting up both services as standalone services independent of those under the DCJ purview was highlighted by one policy expert as a:<br>*"Huge system being setup for DCJ…which is kind of colliding a little bit at the same time" (Their Futures Matter staffer)*<br><br>*Lack of preparedness*<br>Appropriate arrangements were not implemented in the NSW referral system to effectively accommodate for the additional influx of referrals that were expected to be channelled to FFT-CW® and MST-CAN services:<br>*"NSW didn't get the support they needed. The readiness was undercooked. There wasn't a real, a level of readiness in the processes that I could detect" (Intermediary)* |
| **Outcome measures and data collection** | *Administrative burden of DCJ-mandated measures*<br>The overwhelming number of parent and child surveys (n=6) completed by families at FFT-CW® program intake has been criticised:<br>*"They're six different tools…I can't tell you if there's been improvement from the pre and the post. I mean we've only had three families complete the service but I just feel like there's been no visibility around that" (Their Futures Matter staffer)*<br><br>*Confronting nature of tools*<br>Some measures completed at program intake were indicated as confronting and intimidating to vulnerable populations. One such measure is the Composite Abuse Short Form – Revised (CASF-R), which examines domestic violence in female parents and caregivers:<br>*"…You're trying to ask these questions which for one particular tool that we use CASF-R, it is quite confronting and very personal. So how families are reacting to that is usually they're refusing to answer a lot of these questions, or the data is just simply missing because the family's refused" (Their Futures Matter staffer)* |

| | |
|---|---|
| **Workforce, staffing, resourcing and funding issues** | *Timeframes and costs of training*<br>Despite providing quality training, policy experts have highlighted several logistical and monetary issues associated with the organisation of FFT-CW® training in NSW by consultants who reside and operate largely from the United States:<br>*"So that's probably the biggest challenge about training I would say, the scheduling of it, trying to get that balance right. Also, the money required as well, because you're bringing in usually an American trainer, and so there's you know international flight, accommodation and training costs that are associated with that…and that has been really difficult as well" (Their Futures Matter staffer)*<br><br>*Academic qualifications of service providers*<br>Almost unprecedented in DCJ-sponsored child protection programs, the prerequisite that MST-CAN service providers possess a relevant tertiary qualification has consequently minimised the available pool of therapists from which to recruit:<br>*"For MST-CAN, I think that the biggest barrier for them is really just around staffing. It was just really a challenge, and delayed their being able to start, because they're looking for a very specific education level for the therapist positions, and there's just not that many in Australia." (Model purveyor)*<br><br>*Staff recruitment and retainment in rural locations*<br>The high level of academic qualifications required of MST-CAN service providers has made staff recruitment and retainment especially problematic in rural areas in which MST-CAN is delivered:<br>*Some of the barriers that I was seeing was around hiring and it was very difficult for a lot of teams to be able to find qualified applicants and qualified therapists for those positions, especially I think when we go out to some of the more remote areas…I think there's been turnover on certain teams and certain teams just have not been able to fill - have not been able to fill all of the positions yet simply because it's difficult to hire in those areas (Model purveyor)*<br><br>*Under-utilisation of intermediaries and Aboriginal Implementation Support team*<br>In addition to the under-utilisation of OzChild as the local intermediary, AbSec, the Aboriginal Implementation Support Team, has not been sufficiently engaged with and is perceived as under-promoted:<br>*"So it's another one of those things that I can't see where they've been told we [AbSec] weren't available, because we're 100 percent available" (Intermediary)* |

| | |
|---|---|
| **Evidence base and appropriateness for Aboriginal families** | *Lack of cultural acceptability and appropriateness*<br>Some policy and program experts highlighted the need for model purveyors to immerse themselves more deeply in the Australian context to have a better understanding of cultural sensitivity and Aboriginal-specific issues:<br>*"From a cultural standpoint, it is important to spend more time in the jurisdiction that you are going into and understand and think about those cultural adjustments that you might need to think about as we are rolling out" (Their Futures Matter staffer)*<br><br>Ensuring the measures and broader Community Development Team underpinning the FFT-CW® and MST-CAN models comprehensively align with that of Aboriginal families is also recommended:<br>*"There were a number of tools that were culturally, I think, insensitive… data collection tools and probably all of that colliding made it very difficult for them to be set up for success" (Intermediary)*<br><br>*"So we have found that some of the tools that have been used and the terminology within the tools, the descriptors in the tools have not been well accepted well within Aboriginal community and have been seen as offensive" (Their Futures Matter staffer)*<br><br>Making available the evidence of the effectiveness of the FFT-CW® model for Aboriginal families is similarly recommended:<br>*"We're not being given the tools to be able to deliver the program the way it's supposed to be [for Aboriginal families], so we're not actually delivering an evidence-based program" (Intermediary)*<br><br>*Lack of flexibility*<br>Policy experts have stressed the need to increase the flexibility of the intake process whereby families first make contact with the service delivering FFT-CW®:<br>*"There were a number of tools [at program intake] that were culturally, I think, insensitive… data collection tools and probably all of that colliding made it very difficult for them to be set up for success" (Intermediary)*<br><br>The need for flexible and co-designed implementation of the FFT-CW® model with Aboriginal families is similarly stressed:<br>*"Need more around Aboriginal context…no structure or implementation, central implementation body take feedback and co-develop implementation strategies that work in different contexts" (Intermediary)* |

| | |
|---|---|
| | *Engagement phase of FFT-CW®*<br>Extensive efforts were made in the early stages of the FFT-CW® program, namely the engagement phase, to foster a strong therapeutic alliance between Aboriginal families and the therapy team. This rapport is essential to ensuring Aboriginal families effectively complete the later behaviour change and generalisation phases of the FFT-CW® program:<br>*"Relational bonding stuff with Aboriginal families at engagement phase, and you won't get that behaviour change and generalisation without it" (Intermediary)*<br><br>*Role of AbSec*<br>AbSec, as the Aboriginal Implementation Support Team, was considered vital to the effective adaptation of the FFT-CW® program to the NSW context, and both establishing and maintaining positive relationships with Aboriginal service providers and other policy and program experts going forward:<br>*"[AbSec] were also contracted then for that role and they also played a strong role in facilitating that engagement as well locally with the local community and a bit of a link between the service provider and families were required" (Their Futures Matter staffer)*<br><br>*Extensive training in Aboriginal culture*<br>Model purveyors ensuring that MST-CAN staff were adequately trained in Aboriginal culture highlighted the MST-CAN program as conducive and well aligned with Aboriginal families:<br>*"One of the things that you all did nicely was to do some training for us around the Aboriginal culture, and I think we found that MST-CAN is a really good fit with that culture" (Their Futures Matter staffer)*<br><br>*Analytical process*<br>Aboriginal communities praise the analytical process underpinning the MST-CAN program, which conceptualises, prioritises and then systematically addresses the drivers and inhibitors of family goals as part of therapy:<br>*"The Aboriginal community thinks and say very clearly that the way the model thinks about problems fits very well within the Aboriginal community" – re-iterating again it works well because the analytic process thinks about problems in a more circular, rather than linear way" (Their Futures Matter staffer)* |

| | |
|---|---|
| **Adaptation to NSW context** | *Family risk threshold*<br>The risk level of child maltreatment in vulnerable families seen by FFT-CW® service providers is perceived to be greater in Australia that in the United States where the models originated. As one policy expert noted:<br>*"One of the concerns was how the model works well within a domestic violence environment, where the perpetrator's required to be in the home" (Intermediary)*<br><br>*Lack of intermediary support*<br>The local intermediary and the Aboriginal Implementation Support Team never received "cultural awareness training," which was perceived as a barrier to the inclusivity of Aboriginal organisations *(Intermediary)*<br><br>*Camaraderie among FFT-CW® service providers*<br>The strong level of collegiality among service providers united by the common goal of achieving positive outcomes for vulnerable children has been celebrated among policy and program experts, with one emphasising that:<br>*"Service Providers within each cohort are really pretty supportive and have banded together, as opposed to sort of being competitive of nature with each other" (Model purveyor)* |
| **Eligibility criteria of programs** | *Rigidity of criteria*<br>MST-CAN eligibility criteria have been stressed as stringent because it targets a very specific cohort of high-risk families with complex needs:<br>*"This eligibility criteria for MST-CAN is so strict and rigid because when they did the RCT for the CAN adaptation it was based on this was the cohort of families they worked with. So I don't think see how the eligibility criteria can change MST-CAN" (Their Futures Matter staffer)*<br><br>*Lack of clarity surrounding application of criteria*<br>For FFT-CW®, the vagaries of eligibility criteria meant that on occasion, particularly in the early stages of FFT-CW® delivery, ineligible families were being permitted entry into the program:<br>*"In the beginning, it [eligibility criteria] was pretty unclear. I wouldn't say looser criteria, but broader criteria…I would have had probably half [of the] referrals at the beginning that actually weren't appropriate for various reasons" (Their Futures Matter staffer)* |

| | |
|---|---|
| **Relationship with service providers and other stakeholders** | *Differing levels of communication and understanding*<br>While some policy and program experts expressed strong collaboration and communication channels with organisations, others did not. The lack of input of some policy experts in the delivery and adaptation of FFT-CW® to the NSW context in accordance with their roles and responsibilities, has also been highlighted.<br>*"Because we couldn't have any voice, because the lead intermediary didn't want any local folks to say anything, then anything we tried to do was met with "You need to stop doing that, you need to do this, we're in charge, we're running, we have an evidence-based model that we're using"" (Intermediary)*<br><br>*Ongoing guidance from model purveyors and other policy and program experts*<br>Consultancy guidance and support on implementing the FFT-CW® program is received by FFT-CW® and MST-CAN Service Providers on a recurrent and consistent basis, as one stakeholder celebrated:<br>*"You are continually reviewed on how you're implementing the model and you're getting constant support and guidance from the model purveyors and the coaches…both FFT-CW® and MST-CAN both actually have nothing but glowing reports about the consults that get provided" (Their Futures Matter staffer)* |
| **Role of policy expert/nature of contact with the program** | All interviewees clearly stated their nature of contact with the FFT-CW® or MST-CAN program, in addition to their assumed role and responsibilities within their capacity. As previously noted, of the 24 semi-structured interviews, 4 stakeholders were specifically involved with the delivery, implementation and adaptation of the FFT-CW® program, 6 with the MST-CAN program, and an additional 14 stakeholders had a blended role involving both the FFT-CW® and MST-CAN programs. |

| | |
|---|---|
| **Case management processes** | *Balance between case management and therapy*<br>An enduring issue underpinning the FFT-CW® program is balancing the delivery of therapy with that of case management. Presently, the difficulty in managing this twofold approach is ongoing, and the extent to which harmony exists between the two approaches appears to be dependent on individual family circumstances.<br>*"So – but I mean case management is the bigger one, that's been ongoing. It's taken forever and it's still not resolved… I think you will probably get it fairly clear and loud from the service providers as to what that stuff would be – they're the ones on the ground directly dealing with it" (Intermediary)*<br><br>*Specific roles of crisis caseworkers*<br>Unlike FFT-CW®, the specific recruitment of a crisis caseworker in MST-CAN teams ensures that two separate professionals are providing case management and therapy to families receiving MST-CAN:<br>*"I mean, I think one of the, not for MST, but for FFT, one of the larger sort of struggles that came up was striking this balance between case workers and therapists on who was going to do that around case management. That was one of the larger things I saw. For FFT, not MST as much, because MST has specific crisis case workers that sort of handle a lot of those concrete needs" (Model purveyor)* |
| **Implementation facilitators and barriers** | *Community Development Team approach*<br>The Community Development Team (CDT) method on teams-based implementation support has been praised:<br>*"Once the providers were chosen kind of do the procurement process, we provided very specific implementation support to the agencies through a cohort kind of model, that she said, the community development team approach" (DCJ staffer)*<br><br>*Lack of clarity surrounding roles and responsibilities*<br>Greater mutual awareness of the roles of both policy experts and service providers is required to clarify the responsibilities each group has at each stage of the referral process, as one policy expert acknowledges:<br>*"We didn't take enough time in developing the roles, rules and responsibilities, if you will, or what role each member would take when we were working with the local DCJ organisation" (DCJ staffer)*<br>*"Every couple of days or week there would be some new person because we just needed a lot of people to do a lot… maybe we could have done things differently" (Their Futures Matter staffer)* |

| | |
|---|---|
| **Role of intermediaries** | *Training of local intermediary*<br>The local intermediary does not have a clear understanding of its role and responsibility in supporting the implementation of FFT-CW® to NSW because it has not yet received the appropriate Community Development Team (CDT) training to deliver the FFT-CW® program:<br>*“The CDT training was never forthcoming so we haven’t been able to deliver that model of implementation…we didn’t get the training, we sit and listen to those conversations [about how to break down implementation barriers] but we can’t facilitate them”* (Intermediary)<br><br>*AbSec as first point-of-contact*<br>AbSec was perceived as key in providing guidance for services to achieve cultural competence and fidelity, potentially acting as an arbiter in resolving Aboriginal-specific issues:<br>*“…If any of the service providers are working with an Aboriginal family and an issue comes up or they want a case consultation or cultural advice they can go straight to AbSec” (Their Futures Matter staffer)* |
| **Procurement and contracting** | *Block payments*<br>Current personal licencing agreement provisions that service providers are paid upfront a “block payment” for the number of families whom they expect to deliver FFT-CW® are viewed as flawed by some policy and program experts, given these annual quotas are not as yet being met:<br>*“There is a reconciliation, but the reconciliation isn’t against the actual number of kids in the service…If the NGO employs those ten clinicians, and they are ready to go, however, they only have, not the 20 kids but only ten kids in service, because we haven’t referred any, the NGO will still receive the full payment” (Their Futures Matter staffer)*<br><br>*Collaborative referral system setup*<br>Despite acknowledgement that elements of the pre-implementation stage of FFT-CW® and MST-CAN were hasty, a meticulous process involved in the setup of service providers to deliver the programs, with the collaborative assistance of different policy and program experts, was well received:<br>*“So that was quite a detailed process, a quite complicated, involved process in getting the selected service providers setup, what they needed for pre-implementation or to kind of get them ready for implementation…That was a very collaborative process with us and the purveyors and the providers. So I think that was good actually…” (Their Futures Matter staffer)* |

| | |
|---|---|
| **Pre-implementation phase procedures** | *Hasty implementation process*<br>Given the narrow timeframes and external pressures, parts of the implementation and operational processes associated with setting up FFT-CW® have been voiced:<br>*"So I don't think we did it as well as we could, because we were just under a lot of time pressures to get that all completed and done in that timeframe we were required to do from the powers that be. So funny that. So I think there's a lot more operational things we probably could have considered, and would have liked to have done more consultations" (Their Futures Matter staffer)*<br><br>*DCJ readiness to expedite change*<br>The NSW primary child protection agency, DCJ, has been praised by some stakeholders for the aptitude and speed they have shown in their uptake of the FFT-CW® program:<br>*"I've been very impressed with the folks at DCJ and their ability to get this complicated project up and running so quickly. I have seen other implementation projects elsewhere and I have to say that this was the most complicated, quickest, most successful implementation that I have seen. You know, they got a lot done in a very short period of time" (Their Futures Matter staffer)* |
| **Client snapshot/real-time monitoring of families** | *Tracking of families*<br>Ongoing, intricate monitoring of family outcomes as they progress throughout the FFT-CW® and MST-CAN programs has been positively received to date:<br>*"What's also working well is that we're tracking families engagement. So we actually know how many families have been referred; how many have stayed; how many have exited; why they've exited" (Their Futures Matter staffer)*<br><br>*Constant feedback*<br>Having the candour and ability to review the status of families in the FFT-CW® and MST-CAN programs has also been praised:<br>*"I'd have to say that I think the fact that there's a constant check in, like a feedback loop. So it's us checking with the families, reviewing what's working and what's not working, always bringing it back to the table to see how we can do our jobs better and how we can actually teach them to do things better" (Their Futures Matter staffer)* |

| | |
|---|---|
| **Model purveyor training and support** | *Training and ongoing support of program therapists*<br>Staff were comprehensively trained in a wide array of evidence-based techniques and strategies using a strengths-based framework to address the needs of high-risk families:<br>*"We train our therapists in a very broad strategies that are upfront, strengths based and relational. Therapists are given a very concrete set of strategies to do [deliver the program, such as] reframing themes [i.e., cognitions and perceptions of, and interaction patterns with, family members] with families, and given a lot of techniques and tools to make the intervention flexible" (Model purveyor)*<br><br>*Implementation plans*<br>Implementation plans were systematically devised and executed between several policy experts. In particular, model purveyors imparted their invaluable experience in adapting and delivering the MST-CAN program in other jurisdictions to the NSW context:<br>*"So, the New South Wales government was already to the New York Foundling as an agency that had done this work in another jurisdiction, so they were key performance basically for us. So, it was my role to liaise with them to develop the implementation plan, to develop the budget, to identify how many teams in each model we would introduce and how we would introduce them and then to develop the procurement process which selected the suitable provider" (Model purveyor)* |

| | |
|---|---|
| **Nature and structure of FFT-CW® program** | *High acceptance level of FFT-CW® framework*<br>Relative to MST-CAN, the FFT-CW® program appears more conducive to the NSW child protection framework adopted by DCJ. Much of this acceptance is the result of its fidelity, guidance and clarity in objectives, as one policy expert notes the FFT-CW® program has:<br>*"A lot more acceptance within the sector (than MST-CAN)…On the positive side, they appreciate the frameworks. They appreciate the rigour. They appreciate the really good supervision of peers. So peer supervision as well as the consultant supervision and so the intentionality and the focus of the program, clinicians have said they've found that really, really useful" (Their Futures Matter staffer)*<br>*"With FFT-CW® we had a model that sort of fits quite nicely within our service system and applies a similar approach to what we know service providers feel comfortable with" (Their Futures Matter staffer)*<br><br>*Empowerment, strengths-based model*<br>The structure and therapeutic benefits of the FFT-CW® program are unequivocally regarded as one of the key strengths underpinning the program itself:<br>*"The good thing is that it is much more of a capacity building model. It sort of parallels the way in which you work with a family, which is to empower them to find their own solutions and be kind of independent of having to need intervention" (Their Futures Matter staffer)*<br><br>*Positive outcomes for families*<br>As a result of continued therapist support, families are equipped with the skills and autonomy to solve potential issues that may arise in the family home beyond their engagement with the FFT-CW® program:<br><br>*"We've got to really love the success story of a couple therapists working with an Aboriginal family with 8 kids and they're all removed and now they're all back home and everything is really very positive" (Intermediary)* |
