# Supplementary material for: Improving the pragmatic usefulness of the scoring matrix for the Consolidated Framework for Implementation Research (CFIR). A proposal for a more frequency-based approach: The CFIR-f
Source: PLoS One. 2023 Nov 30;18(11):e0295204. doi: 10.1371/journal.pone.0295204 (PMC10688659; doi:10.1371/journal.pone.0295204)
Supplement: S2 Table — (PDF) [file pone.0295204.s002.pdf]

S2 Table. Definitions of CFIR domains and constructs

CFIR domain/construct	Assessment
**1) Innovation**	
A) Innovation source	The program source was external (brought to NSW from the USA). There was strong agreement among all policy experts that:<br>• MST-CAN and FFT-CW® are legitimate therapeutic programs for families in crisis; and<br>• the organisations from which the programs were sourced, and that are supporting their uptake in NSW, are highly credible.
B) Innovation Evidence Base	There was strong agreement among most policy experts that the evidence supporting FFT-CW® and MST-CAN is sound and likely to achieve the desired outcomes. Despite general agreement that evidence strength and quality was an enabler, this agreement was not universal. Specifically, it was noted that:<br>• There is some uncertainty about the evidence (e.g., published literature) for FFT-CW® and MST-CAN more generally.<br>• There may be differences between the target populations from which the existing evidence for FFT-CW® and MST-CAN derives (i.e., juvenile offenders) and the target group in NSW (i.e., all children from 0-17 years for FFT-CW® or 6-17 years for MST-CAN who may or may not have been involved in the juvenile justice sector).<br>• In terms of Indigenous-specific evidence, it was noted that the therapeutic approach ought to be as relevant for Aboriginal families in NSW as for non-Aboriginal families, but that greater adaptation of FFT-CW® or MST-CAN to Indigenous communities is required.

C) Innovation Relative Advantage	There was strong agreement among all policy experts that, relative to previous business as usual services, the provision of a therapeutic approach to families in crisis was a very good idea. There was some perception that FFT-CW® low-track may be less effective than FFT-CW® high-track and MST-CAN because, of the three approaches, it most closely resembled previous business as usual.
D) Innovation Adaptability	Generally, it was perceived by all policy experts that FFT-CW® and MST-CAN are adaptable to NSW, but improvements are necessary on the principle that underpins adaptability: core components and the adaptable periphery. Indeed, on the grounds of optimising fidelity to FFT-CW® and MST-CAN, there was not much room given for this level of adaptation. This generally seems to have been acceptable for non-Aboriginal services, but it appears to be an obvious issue regarding its adaptation to Aboriginal services and families.
E) Innovation Trialability	N/A, given that the independent evaluation assumes the role of investigating the 'trialability' of the program successes, and not the policy experts per se.
F) Innovation Complexity	It was perceived by all policy experts that FFT-CW® and MST-CAN are complex to implement, especially given the structures required to support these programs (for example, the Central Referral Unit (CRU), intermediaries, model purveyors, fidelity/supervision arrangements, training requirements, roles of DCJ caseworkers and service providers). Many of the issues raised in the interviews were focused on this complexity (e.g., the functioning of the CRU and the roles of DCJ caseworkers compared to service providers). It has generally been a barrier to implementation rather than an enabler, perhaps especially for Aboriginal policy experts who had the additional task of ensuring ownership and adaptability to Aboriginal families and services.
G) Innovation Design	Policy experts unanimously perceived the manual-based nature and structure of FFT-CW® and MST-CAN, as well as their quality assurance, accountability and fidelity protocols, as being well-

	assembled and adhered to by all staff. One particularly strong aspect of both programs was the assistance and clinical support provided to service staff by supervisors of program sites and model purveyors. One aspect that a few policy experts perceived as cumbersome was the rigidity of the model – particularly MST-CAN – to be adapted flexibly to individual circumstances of families that were not accounted for in the MST-CAN model.
H) Innovation Cost	Some policy experts stressed the complex financial arrangements entered into between the agency responsible for commissioning the delivery of the programs in the NSW context, and the service providers tasked with their delivery. In addition, a number of concerns were raised regarding the 'block payment' system in which service providers have received renumeration: it was indicated that service providers have been paid a 'lump sum' at the outset of delivering FFT-CW® and MST-CAN equivalent to number of families expected to have received the program at their specific services over a 12-month period. Given that most services have not achieved their anticipated yearly quota, these costs are perceived as sunk, and are unable to be recouped.
**2) Outer Setting**	
A) Critical Incidents	While there were various implementation-related challenges affecting the ability for some services to deliver FFT-CW® or MST-CAN (e.g., delays in referrals being processed and sent to several organisations), and some indication regarding the difficulty establishing both the CRU and contractual agreements for services to deliver the programs, these impediments did not delay the overall implementation of the innovation.
B) Local Attitudes	There is no question that the general focus on improving outcomes for families in crisis, and the specific primary outcomes relating to preventing children from entering out-of-home care, were a shared goal among all policy experts.

C) Local Conditions	The increasing rates of OOHC in NSW, Australia, coupled with the central objective of the NSW Government to preserve and reunify children with their families in an environment of safety and security, has provided an ideal setting to support the ongoing monitoring, investment and delivery of FFT-CW® and MST-CAN.
D) Partnerships & Connections	This was achieved to some extent by the service providers having access to model purveyors and intermediaries for training and support, which was valued by service providers. However, there was a clear lack of cross-service provider networking which may help problem solve more quickly and improve implementation, even though this initial evaluation identified some likely sensitivities (e.g., different service provider organisations having different organisational structures and staffing arrangements).
E) Policies & Laws	An independent review examining the crisis-driven nature of the NSW OOHC system has highlighted the opportunity for policymakers and government to invest in proactive strategies to keep children with their families. Both FFT-CW® and MST-CAN represent family- and strengths-based interventions whose goals are consistent with the recommendations of this review.
F) Financing	While funding of organisations to deliver these innovations is sub-contracted through DCJ, there is indication that additional monetary support may be applicable for some services on a case-by-case basis. It is unclear exactly how these additional funds are subsidised, and under which specific conditions and circumstances.
G) External Pressure	The key external pressures driving the delivery of these programs is pay for delivery, and this approach has clearly achieved the rollout of the programs.
Gi) Societal Pressure	Some service providers highlighted the keen interest government bodies and policymakers have taken to ensure the successes of these innovations, especially given the perceived scrutiny from various community and social groups brought about by the release of the

	independent review on OOHC, and the pressure on them to deliver outcomes for vulnerable children and their families.
Gii) Market Pressure	Market pressure within services was not stressed, however a few noted that some services may be experiencing competitive pressure to receive referrals, and subsequently achieve their quota of families receiving the program at their respective sites. A few also noted that for services delivering FFT-CW® and MST-CAN in locations in which there are several interventions for high-risk families, there is a similar sense of competitive pressure to fill referral quotas and enhance FFT-CW® and MST-CAN uptake.
Giii) Performance Measurement Pressure	From an evaluation perspective, the pay-for-delivery focus has centred on paying for the delivery of the program itself, not the collection of necessary data to evaluate them thoroughly – perhaps a structure could have been set-up, and should be set-up, to monitor the completion of agreed data in real time, and then payments linked to the completion and delivery of those measures/data (as well as the program components).
**3) Inner Setting**	
A) Structural characteristics	This was achieved to some extent by having defined teams to deliver FFT-CW® and MST-CAN with a clear role differentiation from other staff within the service provider organisations. Nevertheless, there was high staff turnover and problems recruiting new staff due to the training timelines and required qualifications. This differentiation was sometimes less clear between the service providers and DCJ caseworkers – this appears to be a communication issue rather than a genuine lack of clarity about these different roles. It also seems possible to decentralise some of the measures (get service providers involved in determining them) which may increase their use and relevance to the programs being delivered.
Ai) Physical Infrastructure	Service provider organisations were all deemed suitable to deliver the innovations overall. As much of FFT-CW® and MST-CAN is delivered at home or a location that is suitable and/or familiar to the

	family receiving treatment, there is less reliance on the physical structure and configuration of the actual service itself.
Aii) Information Technology Infrastructure	The quality of this type of infrastructure was perceived as mixed among stakeholders. While some highlighted the ease with which they could communicate and use technology in their line of work, others deemed the technology of various tools (e.g., intake measures and the Therapist Adherence Measure), and interfaces (e.g., Zoom) at their respective sites as absent and/or problematic.
Aiii) Work Infrastructure	Across most organisations, roles were clearly defined overall. However, in some services, roles (e.g., managers, interventionists) are not filled, and there is a lack of clarity regarding the scope of therapist program delivery (such as the extent to which case management should be prioritised over therapy, and vice versa), as well as high levels of reported staff burnout and turnover. There is also a lack of clarity among some intermediary organisations surrounding how they best support the implementation, adaptation and delivery of the FFT-CW® and MST-CAN programs across NSW.
B) Relational Connections	There is clearly a sense of a team within service providers tasked with delivering FFT-CW® and MST-CAN. Although the quality of relationships fostered between service providers and other stakeholders (e.g., model purveyors, DCJ) have been perceived as somewhat mixed, they are all clearly unified in their objective for preserving children with their families and reducing the number of children who enter OOHC.
C) Communications	Team members are clearly open, in fact seek out and relish, the feedback components on their performance from families, their peers and their supervisors/managers.
D) Culture	The norms and values within all policy experts' organisations are clearly focused on improving outcomes for families in crisis and are well aligned with the introduction of FFT-CW® and MST-CAN. For Aboriginal policy experts however, it appears insufficient attention has been paid to their specific organisational culture of ownership and

	control of their programs. This concept is specific to Aboriginal organisations generally because it is a necessary response to historical issues of loss of control, dispossession, and marginalisation in their own lands. This is the organisational-level manifestation of the issue of adaptability: it is likely FFT-CW® and MST-CAN would be more effectively implemented by Aboriginal policy experts if this cultural component of their organisations was more actively addressed.
Di) Human Equality-Centredness	Families are perceived non-judgementally and as experts of their own lives and circumstances in a dignified and respectful manner from program outset through to completion. The various treatment strategies comprising the innovations allow families to have autonomy and agency at home and in other contexts.
Dii) Recipient-Centredness	The norms and values within all policy experts' organisations are clearly focused on improving outcomes for families in crisis and are well aligned with the introduction of FFT-CW® and MST-CAN.
Diii) Deliverer-Centredness	Service providers perceived the importance of training forums, supervision and ongoing contact with model purveyors to discuss how they can maximise the delivery of programs to families, but also ensure that they are always supported by both their teams and external organisations. The flexibility in the time and location of therapy should also be mutually convenient to both families and therapists.
Div) Learning-Centredness	While there was consensus among stakeholders regarding the importance of the monitoring, safety and appropriate use of data, it is clear this could be improved in several ways: reducing the number of measures at program intake, improving the cultural acceptability and safety of measures for Aboriginal families, and enhancing the integration of various data systems overall.
E) Tension for Change	Not applicable directly in terms of FFT-CW® and MST-CAN specifically, but there is no question all policy experts recognise the need for change.

F) Compatibility	There is clear alignment between the values of FFT-CW® and MST-CAN and those of the individuals and organisations tasked with delivering them.
G) Relative Priority	Clearly all policy experts had a shared perception of the importance of the implementation within the organisation.
H) Incentive Systems	The supervision was well liked and arguably a proxy for 'performance reviews' generally. There was little discussion in the about the other components here (promotions, salary increases, increased stature or respect). These could be considered to drive sustainability over time.
I) Mission Alignment	All key stakeholders and service providers were clearly unified in their objective, consistent with the primary goal of both programs and the over-arching aim of the NSW Government to retain children safety at home, and reduce rates of children at risk of maltreatment who are subsequently removed into OOHC. There was also clear commitment, involvement, and accountability from leaders among the policy experts and managerial patience. Nevertheless, it appears this component could have been improved by greater engagement with leaders in the Aboriginal service providers.
J) Available Resources	All deemed as adequate overall. The only issue we consistently identified was the problem of the timing of training, whereby some stakeholders noted that service staff were burdened by the fact training was staggered at every six months (and reliant on model purveyors coming to New South Wales from the USA to conduct training); there were numerous occasions where they were adequately staffed but could not deliver FFT-CW® or MST-CAN to these families at their sites.
Ji) Funding	Sufficient funding to deliver FFT-CW® and MST-CAN, although it was unclear whether families were able to remain in the programs for longer than the prescribed maximum of 9-months (and whether there was available funding to accommodate for this extension).

Jii) Space	Sufficient physical space to deliver FFT-CW® and MST-CAN across the various sites delivering both programs in NSW.
Jiii) Materials and Equipment	While there were minor discrepancies in the types of resources physically available to some services (e.g., modes of transportation), these were perceived as acceptable on the whole.
K) Access to Knowledge & Information	There was generally large support for this and the interviews clearly identified that it was valued. Nevertheless, the role of the local intermediaries was less clear and information regarding the eligibility and exclusionary criteria of referrals to both FFT-CW® and MST-CAN services was varied among some stakeholders.
**4) Individuals**	
Project Roles	
A) High-level Leaders	Model purveyors had authority for providing high-level implementation feedback to NSW services and held the licensing rights for the FFT-CW® and MST-CAN programs.
B) Mid-level Leaders	There were clearly defined roles among FFT-CW® and MST-CAN managers and supervisors who were considered to have responsibility and oversight for the delivery and implementation of the programs at their respective sites. These individuals reported back to model purveyors who then advised on how program delivery could be further adapted, streamlined and/or tailored to participating families receiving treatment.
C) Opinion Leaders	Expert opinion leaders are indicative of model purveyors /international consultants and program managers. Policy experts highlighted their importance in clarifying any issues therapists may encounter in their line of work – be it fidelity-based, or issues in addressing the problem behaviours of certain families.
D) Implementation Facilitators	Intermediaries at the international (New York Foundling) and local (OzChild, AbSec) level act as a conduit between model purveyors and service providers by providing advice on how to streamline the adaptation, fidelity and cultural appropriateness of programs to both the NSW context and participating families.

E) Implementation Leads	FFT-CW® and MST-CAN managers and supervisors are formally appointed to implement these programs, and act as a primary point-of-contact for the team of therapists, interventionists, or intake workers for whom they have responsibility.
F) Implementation Team Members	DCJ is ultimately responsible for sub-contracting and funding services to deliver FFT-CW® and MST-CAN following successful and competitive tender applications. They are also responsible for liaising with model purveyors to obtain the rights to deliver and adapt both innovations to the NSW context.
G) Other Implementation Support	Policy experts highlighted the importance of therapists and families being subjected to multidisciplinary teams who are each specialised to provide distinct therapeutic interventions.
H) Innovation Deliverers	Service providers comprising managers, supervisions, therapists, interventionists, intake workers and psychiatrics are responsible for the delivery of the innovations
I) Innovation Recipients	Children at high risk of maltreatment and their families are eligible for participation in FFT-CW® and MST-CAN.
Project Characteristics	
J) Need	Children are formally identified as eligible for FFT-CW® and MST-CAN following a rigorous screening process (e.g., the substantiation of maltreatment by child protection authorities and the completion of a Safety Assessment/Risk Assessment (SARA) deeming the child as 'High' or 'Very High' risk of abuse (an eligibility criterion for MST-CAN)).
K) Capability	There are very specific postgraduate and/or tertiary qualifications required of service providers to deliver FFT-CW® and MST-CAN. In addition, therapists receive training on how to administer the innovations at program intake, have access to both their supervisors and model purveyors for feedback and support, and attend additional training, workshops and forums where relevant to enhance their professional development and clinical competencies.

L) Opportunity	Small clinical caseloads ensure therapists can adequately tailor therapy to families to maximise outcomes. At times, the availability of therapists delivering MST-CAN is strained somewhat because of the program technically operating as a 24/7 wraparound service.
M) Motivation	Strong levels of therapist engagement were highlighted for those delivering both FFT-CW® and MST-CAN, in part due to the motivation and desire of service providers to sustain improvements in family communication and interaction patterns, and to ensure the child remains with their parents safely at home, even after their completion of the program.
Implementation	
A) Teaming	There was an international collaboration and a mutual overlap of some tasks (e.g., fidelity support and service provider coaching) by model purveyors and intermediaries to ensure that service providers were maximally supported to deliver the innovations to families. There was also a galvanised effort by all bodies comprising the organisational structure (DCJ, Their Futures Matter, intermediaries and service providers) to implement and adapt both programs to NSW from the outset.
B) Assessing Needs	While the needs of families, community partners and NSW implementation bodies were adequately heard and incorporated into the innovation of both programs overall, greater inclusivity of the perspectives of Aboriginal organisations and DCJ regarding their fidelity and adaptation to NSW is warranted.
Bi) Innovation Deliverers	Policy experts highlighted the positive work of liaising with external stakeholders (particularly model purveyors), particularly during pre-implementation, to subsequently maintain a system of strong quality assurance, accountability and fidelity going forward. Nonetheless, some stakeholders noted that further improvements to the data measures (e.g., TAM) and educational requirements of service staff, especially in rural and remote NSW locales, is warranted, and that these perspectives have not yet been embedded in program delivery.

Bii) Innovation Recipients	While some policy experts acknowledged there was sufficient consultation with Aboriginal stakeholders to ensure both programs were culturally appropriate for Aboriginal families, others would have preferred greater co-design and ongoing and sustained partnership beyond pre-implementation.
C) Assessing Context	Among Aboriginal services, in particular, there were mixed perspectives regarding execution; whereas the implementation of MST-CAN was reported as effective, FFT-CW® was hindered in its delivery by a number of process (e.g., engagement with cultural mentors) and structure-based issues (e.g., lack of trauma-informed approach underpinning the program).
D) Planning	The planning stages of FFT-CW® and MST-CAN implementation were in places, poorly conducted. The financial aspects of FFT-CW® and MST-CAN contracts – whereby services were initially paid a lump sum payment based on their expected annual benchmark of families engaging in either program at their site – was poorly planned, as was the geographical location of a number of FFT-CW® and MST-CAN services.
E) Tailoring Strategies	While some policy experts perceived the innovations as not appropriately accommodating for the risk thresholds of families in NSW, others deemed the need to amend eligibility criteria to ensure they are available to as many families whose children are at risk of maltreatment as possible (e.g., relaxing the eligibility criteria of FFT-CW® to allow families of different risk thresholds to be referred to the innovation).
F) Engaging	While clearly defined frameworks are not in place to sustain engagement with both service providers and especially families, policy experts acknowledged that great efforts are made to include the perspectives of families in their modus operandi, and to ensure that this feedback is actively communicated back to intermediaries and model purveyors in real-time, and on an ongoing basis.

Fi) Innovation Deliverers	Competitive remuneration rates were offered to service providers to deliver and implement the programs at their respective sites, and there was a general alignment of the innovations with the broader objectives of family and child wellbeing underpinning the other programs delivered by service providers at their sites.
Fii) Innovation Recipients	Families provide feedback via several metrics associated with the delivery of the innovations, such as the TAM. A few policy experts also noted the potency of family feedback and testimonials influencing how the innovations are delivered (e.g., ensuring that Aboriginal female therapists deliver therapists exclusively to Aboriginal mothers).
G) Doing	Overall, execution for non-Aboriginal services was somewhat adequate from a fidelity, but not outcome perspective. Strong quality assurance and weekly supervision with consultants ensured therapists were adhering to the manual-based treatment of either program.
H) Reflecting and evaluating	Policy experts highlighted evaluation and reflection as effective on a service-specific level, given the extensive contact between therapists and supervisors, and therapists and consultants. However, when considering the programs holistically, policy experts stressed the lack of consistency and integration of relevant stakeholders.
Hi) Implementation	An independent evaluation was conducted of the processes and outcomes underpinning the enablers and barriers associated with the implementation of FFT-CW® and MST-CAN.
Hii) Innovation	Policy experts highlighted that model purveyors continually update and tailor the innovations to ensure their fidelity is maintained, and that they are fit-for-purpose for families who require them the most. Although this always occurs after the evaluation of both programs when they have been newly introduced and examined in a different jurisdiction or context, the successes of the programs are assessed on an ongoing basis.
I) Adapting	Despite the fidelity and rigidity of some FFT-CW® and MST-CAN eligibility criteria, adaptations to both innovations were made to

	increase engagement and foster rapport with Aboriginal families (Cultural Consultants), as well as the creation of the local intermediary (OzChild) and Aboriginal Implementation Support team (AbSec) to assist in streamlining the adaptation and implementation of FFT-CW® and MST-CAN in NSW. The successes of both these initiatives were perceived as largely mixed.
