# Supplementary material for: Improving the pragmatic usefulness of the scoring matrix for the Consolidated Framework for Implementation Research (CFIR). A proposal for a more frequency-based approach: The CFIR-f
Source: PLoS One. 2023 Nov 30;18(11):e0295204. doi: 10.1371/journal.pone.0295204 (PMC10688659; doi:10.1371/journal.pone.0295204)
Supplement: S1 Appendix — (PDF) [file pone.0295204.s003.pdf]

S1 Appendix. Evaluation questions to assess adaptation and implementation of FFT-CW® and MST-CAN in NSW, Australia

1. How is the referral system currently functioning?

2. To what extent are families being retained in the program?

3. Has adaptation of the models for Aboriginal/non-Aboriginal families been adequate?

4. Are the contracting and financial arrangements effective and efficient?

5. How is the data collection system currently functioning?

6. Are the models being delivered as planned (with fidelity)?
