# Supplementary material for: Improving the pragmatic usefulness of the scoring matrix for the Consolidated Framework for Implementation Research (CFIR). A proposal for a more frequency-based approach: The CFIR-f
Source: PLoS One. 2023 Nov 30;18(11):e0295204. doi: 10.1371/journal.pone.0295204 (PMC10688659; doi:10.1371/journal.pone.0295204)
Supplement: Attachment — Submitted filename: Response to Reviewers.docx [file pone.0295204.s004.docx]

**Journal requirements:**

| Journal requirements | My response |
| --- | --- |
| 1. Please ensure that your manuscript meets PLOS ONE's style requirements, including those for file naming. | I can confirm the manuscript meets PLOS ONE’s style requirements, including those for file naming. |
| 2. Thank you for stating the following in the Competing Interests section:  “Anthony Shakeshaft was the lead investigator on a competitive tender (FACS.17.266) awarded by the NSW Government Department of Family and Community Services (now Department of Communities and Justice) to evaluate the Functional Family Therapy – Child Welfare [FFT-CW®] and Multisystemic Therapy for Child Abuse and Neglect [MST-CAN] programs in New South Wales, Australia (2018-2020). George Economidis was employed part-time at the National Drug and Alcohol Research Centre as a Project Coordinator for the FFT-CW® and MST-CAN program evaluation from May 2018 to August 2020.”  Please confirm that this does not alter your adherence to all PLOS ONE policies on sharing data and materials, by including the following statement: "This does not alter our adherence to PLOS ONE policies on sharing data and materials.” (as detailed online in our guide for authors <http://journals.plos.org/plosone/s/competing-interests>).  If there are restrictions on sharing of data and/or materials, please state these. Please note that we cannot proceed with consideration of your article until this information has been declared.  Please include your updated Competing Interests statement in your cover letter; we will change the online submission form on your behalf. | I have now included the statement “this does not alter our adherence to PLOS ONE policies on sharing data and materials” in both the updated Competing Interests section of the Declarations form and the Competing Interests statement in my cover letter. |
| 3. Thank you for stating the following in the Acknowledgments Section of your manuscript:  “The research team acknowledges and thanks all policy experts and service providers who participated in data collection for this study. We acknowledge the contribution of Dr. Paula Jops and Dr. Hueiming Liu to conducting interviews with some of the policy experts in this study. NDARC receives funding from the Australian Government’s Department of Health.”  We note that you have provided funding information that is currently declared in your Funding Statement. However, funding information should not appear in the Acknowledgments section or other areas of your manuscript. We will only publish funding information present in the Funding Statement section of the online submission form.  Please remove any funding-related text from the manuscript and let us know how you would like to update your Funding Statement. Currently, your Funding Statement reads as follows:  “GE was supported by an Australian Government Research Training Program (RTP) Scholarship via the University of New South Wales (UNSW), Sydney, Australia, and a Higher Degree Research scholarship from the National Drug and Alcohol Research Centre (NDARC), UNSW, Sydney. The evaluation of the MST-CAN program in New South Wales, Australia (2018-2020) was funded via a competitive tender process (FACS.17.266) by the NSW Government Department of Family and Community Services (now Department of Communities and Justice). Their Futures Matter and Department of Communities and Justice staff provided feedback on the evaluation plan, participated in interviews as key stakeholders in this evaluation, and reviewed the manuscript.”  Please include your amended statements within your cover letter; we will change the online submission form on your behalf. | I have removed any reference to funding information in the Acknowledgements section, or in any other section of the revised manuscript.  I have provided updated wording at the bottom of the cover letter regarding my updated funding statement and thank PLOS ONE for updating the online submission form on my behalf:  “GE was supported by an Australian Government Research Training Program (RTP) Scholarship via the University of New South Wales (UNSW), Sydney, Australia, and a Higher Degree Research scholarship from the National Drug and Alcohol Research Centre (NDARC), UNSW, Sydney. NDARC is supported by funding from the Australian Government Department of Health and Aged Care under the Drug and Alcohol Program. The evaluation of the FFT-CW^®^ and MST-CAN programs in New South Wales, Australia (2018-2020) was funded via a competitive tender process (FACS.17.266) by the NSW Government Department of Family and Community Services (now Department of Communities and Justice). Their Futures Matter and Department of Communities and Justice staff provided feedback on the evaluation plan, participated in interviews as key stakeholders in this evaluation, and reviewed the manuscript”. |
| 4. Please include captions for your Supporting Information files at the end of your manuscript, and update any in-text citations to match accordingly. Please see our Supporting Information guidelines for more information: <http://journals.plos.org/plosone/s/supporting-information>. | I have now added captions for my Supporting Information files at the end of the manuscript and updated in-text citations to match accordingly. |
| 5. Please review your reference list to ensure that it is complete and correct. If you have cited papers that have been retracted, please include the rationale for doing so in the manuscript text, or remove these references and replace them with relevant current references. Any changes to the reference list should be mentioned in the rebuttal letter that accompanies your revised manuscript. If you need to cite a retracted article, indicate the article’s retracted status in the References list and also include a citation and full reference for the retraction notice. | I can confirm that my reference list is complete and correct. No cited papers have been retracted since this manuscript was peer-reviewed. However, with the additional references noted by the reviewer in their comments below, the reference order has changed since the initial review of this manuscript.  In addition, please note that we have included one reference that we anticipated would be published by now (reference number 18). However, we are still awaiting final confirmation from that journal, so I have edited the wording of this reference to:  Economidis G, Farnbach S, Falster K, Eades AM, Shakeshaft A. Enablers and barriers to the implementation of Multisystemic Therapy for Child Abuse and Neglect (MST-CAN) into the routine delivery of child protection services. Children and Youth Services Review. Under editorial review.  I trust that is reasonable, and I can forward the confirmation of this paper being accepted as soon as possible. Alternatively, we can edit the relevant section in the paper to remove this reference. |

**Review Comments to the Author**

Please use the space provided to explain your answers to the questions above. You may also include additional comments for the author, including concerns about dual publication, research ethics, or publication ethics. (Please upload your review as an attachment if it exceeds 20,000 characters)

Reviewer #1: Thank you for the opportunity to review this paper, “Improving the pragmatic usefulness of the scoring matrix for the Consolidated Framework for Implementation Research (CFIR). A proposal for a more frequency-based approach: the CFIR-f.”

This paper reports and proposes a pragmatic, structured, frequency-based approach to applying the Consolidated Framework for Implementation Research +2/-2 scoring system. Given the number of domains, constructs and subconstructs (n=67) that the updated CFIR contains, innovative and pragmatic ways to analyse, and interpret, data obtained from applying the updated CFIR (and implementation theories, models and frameworks in general) is important. At the same time the authors identify several valid issues associated with previously published criteria to support researchers and practitioners apply the +2/-2 scoring system.

Specific questions and critiques follow in the order that they appear in the manuscript.

***These specific questions and critiques are listed and addressed in the table on the next page:***

| **Reviewer’s comment** | **My response** |
| --- | --- |
| Abstract Line 61: The CFIR-f engenders a list of themes, ranked by key experts according to their implied importance, to inform policymakers and services about priorities for action. I have serious concerns regarding this conclusion, is it not more accurate to conclude that the proposed scoring system generates a list of barriers and facilitators, ranked by frequency, rather than ‘according to their implied importance’. | Yes, we agree and have now revised the final sentence of the Abstract in line with the reviewer’s suggestion that this study more strongly reflects a list of enablers and barriers based on the frequency with which they were identified. This final sentence of the Abstract now reads as follows (lines 52-55):  “By specifically utilising the frequency with which these barriers and enablers are identified, the CFIR-f engenders a list of ranked themes that service providers and policymakers can use to inform their decisions about program modification and implementation”. |
| Background Line 67, missing references: Programs that simultaneously intervene at the individual, community and societal level are increasingly being utilised, both to optimise their effectiveness and improve the sustainability with which they are integrated into real-world service delivery. | I have now provided two references to this effect on Lines 58-61: Johnston et al. (2013) **[1]**; Knight et al. (2017) **[2].** (Please note that these additional references have now changed the ordering of references in the remainder of the manuscript – for example, in the reviewer’s next comment regarding Line 72, the relevant example originally referenced as [1, 2] is now referenced as [3, 4].)  The citations for both of these references can be found in the reference list of the manuscript and are as follows:  Johnston L, Doyle J, Morgan B, Atkinson-Briggs S, Firebrace B, Marika M, et al. A review of programs that targeted environmental determinants of Aboriginal and Torres Strait Islander health. International Journal of Environmental Research and Public Health. 2013;10(8): 3518-3542. [doi: 10.3390/ijerph10083518](https://doi.org/10.3390/ijerph10083518).  Knight A, Shakeshaft A, Havard A, Maple M, Foley C, Shakeshaft B. The quality and effectiveness of interventions that target multiple risk factors among young people: A systematic review. Australian and New Zealand Journal of Public Health. 2017;41(1): 54-60. [doi: 10.1111/1753-6405.12573](https://doi.org/10.1111/1753-6405.12573).  In addition, I have slightly re-worded this statement noted by the reviewer to read as follows: “Programs that simultaneously respond to harms that emanate from the individual, community and societal level are increasingly being utilised, both to optimise their effectiveness and improve the sustainability with which they are integrated into real-world service delivery [1, 2]”. |
| Line 72: Identifying the enablers of, and barriers to, the delivery of multi-level programs at an early stage of their implementation is especially important because it is a particularly volatile stage in the program delivery process [1, 2]. I think it is important to add that identifying implementation determinants at an early stage can help inform implementation efforts (e.g., adaptations and modifications to the intervention and/or implementation strategy). | We agree this is an important point. I have now added a sentence expanding on the importance of identifying enablers and barriers to program delivery during the early stages of implementation (Line 63-69):  “Identifying the enablers of, and barriers to, the delivery of multi-level programs at an early stage of their implementation is especially important because it is a particularly volatile stage in the program delivery process [3, 4]. Moreover, identifying the factors that influence the early implementation of a program creates the opportunity for the enablers to be leveraged and the barriers to be modified, to optimally tailor the program to the context in which it is being delivered before the processes that support its implementation become entrenched” (Ronto et al., 2020) [5] |
| Line 77, missing reference: One such framework is the Consolidated Framework for Implementation Research (CFIR) | Thank you. I have added the following reference (Line 70-71): Damschroder et al. (2009) **[6]**  Damschroder, L. J., Aron, D. C., Keith, R. E., Kirsh, S. R., Alexander, J. A., & Lowery, J. C. (2009). Fostering implementation of health services research findings into practice: a consolidated framework for advancing implementation science. *Implementation Science*, *4*(1), 1-15. <https://doi.org/10.1186/1748-5908-4-50> |
| Line 87: In brief, implementation evaluators would typically convene a group of people involved in the delivery of a program and, using an established qualitative method (e.g., semi-structured interviews), ask a series of questions that align with some or all of CFIR’s constructs. This is one way CFIR can be applied, but I’m not convinced it can be described as the ‘typical’ way. I suggest the authors read/cite the following paper and provide a more accurate summary of how CFIR can has been used: Kirk, M.A., Kelley, C., Yankey, N. et al. A systematic review of the use of the Consolidated Framework for Implementation Research. Implementation Sci 11, 72 (2015). <https://doi.org/10.1186/s13012-016-0437-z> | Thank you, the citation provided by the reviewer has been most helpful in enhancing my description regarding the application of the CFIR, which now reads as follows (Lines 79-87):  “In brief, evaluators have used the CFIR to measure the impact of a wide range of variables on the implementation of a program by using data generated by both qualitative and quantitative research methods. From a qualitative perspective, evaluators have used the CFIR as a framework, or ‘checklist’, for coding information collected using standardised qualitative techniques, such as focus group discussions and semi-structured interviews [10]. From a quantitative and mixed-methods perspective, evaluators have applied ratings, or rankings, to the CFIR’s constructs based on the results of surveys or questionnaires [7, 14]. It is worth noting that most studies have only used the CFIR to inform their data analysis rather than their data collection [10]”. |
| Line 96: The answers to each of these questions would then be analysed using a standardised thematic analysis, and each theme is allocated a score by the evaluators ranging from +2 to -2: themes rated +1 or +2 are classified as enablers; themes rated -1 or -2 are classified as barriers; and themes rated as 0 are classified as being neither an enabler nor a barrier to implementation [4, 12]. Related to the previous point, I think it needs to be made clear that the +2/-2 scoring system is frequently applied. | Yes, agreed. I have now revised this sentence in line with the recommended wording provided by the reviewer (Lines 93-96):  “Evaluators then frequently apply a scoring system, whereby each theme is allocated a score from +2 to -2: themes rated +1 or +2 are classified as enablers; themes rated -1 or -2 are classified as barriers; and themes rated as 0 are classified as being neither an enabler nor a barrier to implementation [7, 16]”. |
| Line 101: I suggest rewording ‘actual barriers and enables’ to ‘more influential barriers and enablers’: The more extreme scores (i.e., +2 and -2) are interpreted as actual barriers and enablers, while +1 and -1 are interpreted as being generally influential. The word ‘actual’ implies that barriers and enables scored +1 or -1 are not ‘actual’ barriers and enablers, which is not the cases. | Yes, this is very helpful. I have amended this sentence to now read “The more extreme scores (i.e., +2 and -2) are interpreted as more influential barriers and enablers, while +1 and -1 are interpreted as being generally influential” (Lines 96-98). |
| Methods  Table 1 is very useful and clear | No response required. |
| Results Table 2 title add 'and neither enablers or barriers’. Please make it clear in the column heading, what the number in brackets in the first column represent. E.g. ‘Technical, logistical and referral challenges (n=19)’. This is not obvious. | The title of Table 2 now reads: “The 16 themes identified as enablers and barriers, and neither enablers or barriers, to the implementation of MST-CAN and FFT-CW^®^ in NSW”.  I have also added further information in Table 2 describing what the number in brackets in the first column represents:  “Themes (n=total number of participants who identified each theme)”. |
| Line 224: The logic is to provide services, funders and policymakers with a list of barriers and enablers that are weighted according to their importance, as determined by the frequency with which they are mentioned by the experts participating in each group. I disagree with the following part of this statement: ‘a list of barriers and enablers that are weighted according to their importance’. I strongly suggest changing to ‘a list of barriers and enablers by the frequency with which they are mentioned…’ This issue arises throughout the paper, please review and amend each statement accordingly (e.g., Line 387: the CFIR-f allocated a continuous score to each theme which can be interpreted as a list of themes prioritised by experts). The themes were not ‘prioritised’ by experts | Thank you, this is an excellent suggestion. I have addressed this comment in relation to the specific examples provided by the reviewer and by carefully searching the rest of the manuscript for other instances of this wording. In relation to the specific comments, I have made the following two changes:  “The logic is to provide services, funders and policymakers with a list of barriers and enablers determined by the frequency with which they are mentioned by participants across each of the four expert groups” (Line 206-209).  “In contrast to the original CFIR scoring method, the CFIR-f allocated a continuous score to each theme. This generated a list of themes, ranked by the frequency with which each theme was identified by a range of experts involved in the delivery of the program. Synthesising the enablers and barriers in this way provides policymakers with an objective method for prioritising their decisions about how the implementation of a program might be most effectively and efficiently improved” (Lines 354-359).  In relation to the rest of the manuscript, I have removed all other references to the enablers and barriers being weighted according to their importance, as well as any reference to themes prioritised by experts. Given the reference to ‘importance’ and ‘priority’ has been indicated on 9 and 19 occasions throughout the manuscript, respectively, I have not provided each of these revisions in this rebuttal letter (however, the amendments made in each of these instances, which have been modified to reflect the revised wording in my response to the two specific examples provided by the reviewer above, are indicated in the updated version of the manuscript with tracked changes). |
| Discussion Line 443. A major limitation to the CFIR-f approach is that identified barriers and enablers are ranked and ‘prioritised’ based solely on frequency, rather than frequency AND strength of influence on implementation. I don’t think the authors go far enough in highlighting and discussing the implications of this limitation. I think it would be useful to compare this approach to the original CFIR scoring approach, that determines strength of influence on implementation by considering a number of factors, including level of agreement among participants, strength of language, and use of concrete examples. Would a matrix approach, considering strength and frequency be the most desirable scoring approach? Perhaps this is something to highlight that should be explored in future research. | Thank you. Again, this is very helpful. I have now added the following new text to the ‘Methodological considerations’ section in the Discussion of the manuscript to highlight this issue noted by the reviewer, especially the potential to more effectively synergise the proposed CFIR-f with the some of the defining characteristics of the original CFIR: (Lines 449-487):  “Another consequence of using the 50% cut-off threshold for identifying themes was that none of the themes identified by the DCJ participants were classified as enablers or barriers (because less than 50% of the DCJ group identified these themes). Although this suggests that there was less consensus regarding implementation issues among DCJ participants, relative to the other three policy and program expert groups, it may also highlight that further consideration could be given to how best to determine the level at which this cut-off threshold is set, including the idea that it might vary according to the circumstances of each evaluation. Alternatively, the cut-off threshold for identifying themes could be determined by using different, or more than one, criteria to reduce the reliance on one frequency-based criterion. For example, the threshold could be defined as including all enablers and barriers that were identified by at least 50% of participants and the most frequently cited enabler and barrier from any group whose views are excluded by the 50% threshold.  In addition, this initial version of the CFIR-f only considers the extent to which each enabler and barrier influences the implementation of a program through the assumption that the more frequently identified themes may be more influential. Future research could test the validity of this assumption. While the CFIR does not use this frequency-based approach (e.g., it only allocates a score of +2 or -2 to any theme identified by at least two participants), it does attempt to capture how strongly different enablers and barriers are likely to have influenced implementation by using complementary indicators, such as the level of agreement among participants, strength of language and the use of concrete examples [7]. However, a limitation of these indicators, and a key reason for the development of the CFIR-f, is their reliance on the evaluation team to interpret concepts such as “level of agreement” and “strength of language”. Moreover, these indicators ultimately rely on the same type of assumption as the CFIR’s frequency-based method in interpreting their pragmatic meaning: the use of “concrete examples” may reflect the relative simplicity of articulating a particular enabler or barrier, for instance, rather than the perceived strength of its influence on the implementation of a program.  A potential solution that could be tested in future research is the adoption of a matrix approach that incorporates both the frequency-based process of the CFIR-f and a less subjective method of capturing the relative impact of different enablers and barriers on the implementation of a program. The nominal group technique (NGT) method [25], for example, is a consensus-based approach to identifying priorities that the authors used in the evaluation of the FFT-CW^®^ and MST-CAN programs in NSW [18, 19]. It may be possible to combine the thematic analysis approach in this study with the NGT method in either a two-step process (e.g., semi-structured interviews and thematic analysis, followed by the ranking of themes using NGT in a subsequent session) or a one-step process if it becomes possible to harness artificial intelligence (AI) technology to perform thematic analysis with sufficient rapidity and accuracy”.  Given our comprehensive response to this comment by the reviewer above, please note that we have subsequently removed an existing small paragraph (Lines 451-455 in the marked up/tracked change version of the manuscript) because it is now too repetitive and most likely redundant. |
| Line 457. I disagree that it is reasonable to conclude ‘that technical, logistical and referral challenges [score -11] is regarded by experts as being about three times more problematic than the need for clarification and amendment of the eligibility criteria of the programs ([score -3]).’ At best, the authors can state that technical, logistical and referral challenges were identified about 3 times more frequently as a barrier to implementation than the needs for clarification and amendment of the eligibility criteria of the programs. Frequency and the degree to which a barrier is ‘problematic’ are different. How is the CFIR-f more pragmatic than the CRIF approach? It would be useful for the authors to define what they mean by ‘pragmatic usefulness’. | Thank you. I have revised this sentence in line with the recommended wording provided by the reviewer:  “In this study, for example, it is reasonable to conclude that technical, logistical and referral challenges [score -11] were identified about three times more frequently as a barrier to implementation than the need for clarification and amendment of the eligibility criteria of the programs ([score – 3])” (Lines 419-422).  In addition, in the same section, I have amended my description of pragmatic usefulness (Lines 416-438): to read:  “Furthermore, the relative advantage of the CFIR-f is that it generates a frequency-based list of enablers and barriers for policymakers to action based on continuous (not ordinal) scores from the perspectives of key stakeholders who have been actively engaged in the delivery of a program. In this study, for example, it is reasonable to conclude that technical, logistical and referral challenges [score -11] were identified about three times more frequently as a barrier to implementation than the need for clarification and amendment of the eligibility criteria of the programs ([score -3]). Using the original CFIR framework, both these themes would be allocated the same score of -2 for each expert group who identified them as barriers, irrespective of whether two or more stakeholders in each group articulated these themes as such. Thus, the original CFIR framework does not provide policymakers with sufficiently actionable information, that is, information that would be useful in assisting their decision-making about which of these barriers to prioritise to achieve relatively greater improvement in the ongoing implementation of a program.  In the real-world context of policymakers and service providers having limited resources and time, the continuous scores generated by the CFIR-f enhances the pragmatic usefulness of the results of an implementation evaluation. Specifically, it generates information that originates directly from the experiences of key stakeholders who have been engaged in the implementation of a program, it is less susceptible to evaluator bias, and it presents a list of enablers and barriers that are ranked by the frequency with which they were identified. Generating and presenting information in this way makes that information more actionable for policymakers and service providers in determining the order in which barriers should be addressed, and enablers should be leveraged, to optimally improve implementation”. |

PLOS authors have the option to publish the peer review history of their article. If published, this will include your full peer review and any attached files. **Yes, I agree to publish the peer review history of my article.**
